# Hippocampal volume, *FKBP5* genetic risk alleles, and childhood trauma interact to increase vulnerability to chronic multisite musculoskeletal pain

**DOI:** 10.1038/s41598-022-10411-9

**Published:** 2022-04-20

**Authors:** Jarred J. Lobo, Lizbeth J. Ayoub, Massieh Moayedi, Sarah D. Linnstaedt

**Affiliations:** 1 Institute for Trauma Recovery, University of North Carolina, Campus Box #7010, Chapel Hill, NC 27599-7010 USA; 2 Department of Anesthesiology, University of North Carolina, Chapel Hill, NC USA; 3 Centre for Multimodal Sensorimotor and Pain Research, Faculty of Dentistry, University of Toronto, Toronto, ON Canada; 4 University of Toronto Centre for the Study of Pain, Toronto, ON Canada; 5 Division of Clinical and Computational Neuroscience, Krembil Brain Institute, Toronto Western Hospital, University Health Network, Toronto, ON Canada; 6 Department of Dentistry, Lunenfeld-Tanenbaum Research Institute, Mount Sinai Hospital, Toronto, ON Canada; 7 Centre for Multimodal Sensorimotor and Pain Research, Faculty of Dentistry, University of Toronto, 123 Edward Street, Suite 501B, Toronto, ON M5G 1G6 Canada

**Keywords:** Genetics of the nervous system, Genetics, Neuroscience, Psychology

## Abstract

Chronic multisite musculoskeletal pain (CMP) is common and highly morbid. However, vulnerability factors for CMP are poorly understood. Previous studies have independently shown that both small hippocampal brain volume and genetic risk alleles in a key stress system gene, *FKBP5*, increase vulnerability for chronic pain. However, little is known regarding the relationship between these factors and CMP. Here we tested the hypothesis that both small hippocampal brain volume and *FKBP5* genetic risk, assessed using the tagging risk variant, *FKBP5*rs3800373, increase vulnerability for CMP. We used participant data from 36,822 individuals with available genetic, neuroimaging, and chronic pain data in the UK Biobank study. Although no main effects were observed, the interaction between *FKBP5* genetic risk and right hippocampal volume was associated with CMP severity (β = −0.020, p_raw_ = 0.002, p_adj_ = 0.01). In secondary analyses, severity of childhood trauma further moderated the relationship between *FKBP5* genetic risk, right hippocampal brain volume, and CMP (β = −0.081, p = 0.016). This study provides novel evidence that both *FKBP5* genetic risk and childhood trauma moderate the relationship between right hippocampal brain volume and CMP. The data increases our understanding of vulnerability factors for CMP and builds a foundation for further work assessing causal relationships that might drive CMP development.

## Introduction

Up to one-third of individuals globally suffer from chronic pain in their lifetime^1–3^. Of those individuals experiencing chronic pain, a substantial subset have musculoskeletal pain in multiple sites across the body^4–6^. Such chronic multisite musculoskeletal pain (CMP) is highly severe^7,8^, is one of the most common reasons for years lived with disability^9–11^, contributes to high health care costs^12,13^, and can lead to opioid addiction/abuse^14,15^. Despite the substantial burden that CMP has on the individual and society, vulnerability factors for CMP and the interplay between these factors are poorly understood. This lack of understanding precludes the development of effective diagnostic tools and impedes the identification of promising targets for therapeutic intervention.

Increasing evidence indicates that two highly influential vulnerability factors for chronic musculoskeletal pain are smaller hippocampal volumes^16^ and the presence of one or more genetic risk alleles in a key stress system gene, *FKBP5*^17,18^. While the data supporting these vulnerability factors originate from independent studies, multiple lines of evidence suggest that these two risk factors are related and might interact to influence CMP development. First, both the hippocampus and *FKBP5* are involved in the regulation of the physiological response to stress^19–23^. Second, these two vulnerability factors have been shown to influence chronic pain states in both human and animal studies^24–28^. Third, *FKBP5* gene expression increases in the hippocampus in response to stress (which can lead to decreased glucocorticoid receptor sensitivity)^29,30^. Fourth, studies assessing neuropsychiatric disorders that often overlap with CMP, such as depression and anxiety, have shown (albeit in modest sample sizes) that genetic/molecular risk in *FKBP5* influences hippocampal brain volume and that these factors together are associated with neuropsychiatric pathology^31–34^. Altogether this data suggests that small hippocampal volume and risk alleles in *FKBP5* might interact to influence CMP vulnerability.

In addition to brain anatomy and *FKBP5* genetics, another previously identified vulnerability factor for CMP is early life adversity via childhood trauma^35,36^. Previous data indicates that individuals with a history of trauma have increased rates of CMP^37–39^. Further, related to *FKBP5* genetic risk and hippocampal volumes as vulnerability factors for CMP, individuals with a history of trauma and adverse sequelae have been shown to have smaller hippocampal volumes^40,41^ and genetic/molecular risk in the *FKBP5* locus^42–44^. Therefore, it is possible that the combination of all three vulnerability factors — the stress of childhood trauma, *FKBP5* genetic risk, and hippocampal volume — increases risk for CMP.

In the current study, we tested the registered hypotheses^45^ that (1) hippocampal volume is associated with CMP severity, (2) *FKBP5* genetic risk via rs3800373 is associated with CMP severity, and (3) that these two vulnerability factors interact to influence CMP, i.e. *FKBP5*rs3800373 moderates the relationship between hippocampal volume and CMP severity. These hypotheses were tested using neuroimaging, genetic, and musculoskeletal pain data collected from 36,822 participants enrolled in the UK Biobank study. In secondary analyses, we assessed whether childhood trauma further moderated the relationship between hippocampal volume, *FKBP5*rs3800373, and CMP. Together, this work contributes to our understanding of vulnerability factors for CMP.

## Results

### Participants

The UK Biobank cohort consisted of 502,487 participants, of which 36,822 participants had genetic, brain imaging, and chronic pain data and were included in the current study. Characteristics of participants (n = 36,822; 54% women) are shown in Table 1. The majority of individuals self-identified as White (97%), were more than sixty years of age, had at least some college education, and were overweight (BMI > 25).

**Table 1:** Characteristics of UK Biobank participants with available data for primary study analyses (n = 36,822).

**Characteristic**	
Age, years, mean (SD)	63.6 (7.5)
Women, n (%)	19,288 (52.4)
**Education, n (%)**	
NVQ, HND, HNC, or equivalent, or other professional qualification	6281 (17.1)
O levels, GCSEs, CSEs, or equivalent	7938 (21.6)
A levels, AS levels, or equivalent	4517 (12.3)
College or university degree	17,967 (48.8)
**Self-identified race/ethnicity**	
White	35,770 (97.1)
Asian or Asian British	377 (1.0)
Black or Black British	213 (0.6)
Multiethnic or other	462 (1.3)
BMI, mean (SD)	26.5 (4.4)
Current smoker, n (%)	1272 (3.5)

*SD* standard deviation, *n* sample size,* NVQ* national vocational qualification, *HND* higher national diploma, *HNC* higher national certificate, *O levels* ordinary levels, *GCSE* general certificate of secondary education, *CSE* certificate of secondary education, *A level* advanced levels, *AS level* advanced subsidiary levels, *BMI* body mass index.

### *FKBP5*rs3800373 genetic risk and hippocampal brain volume were not directly associated with CMP in UKBB participants

Our three primary hypotheses, as submitted in our UK Biobank proposal^45^, were that (a) hippocampal brain volume, (b) *FKBP5*rs3800373 genetic risk, and c) the interaction between these two biological factors are associated with CMP. To test these hypotheses, we first examined the relationship between *FKBP5*rs3800373 genetic risk and CMP severity, defined by the number body sites with musculoskeletal pain for ≥ 3 months. In the UK Biobank cohort used here, 32.7% (n = 12,038) of individuals reported one or more sites of CMP, with the average number of sites of CMP equal to 0.48 (SD = 0.81) (Supplementary Table 1). Using linear regression modeling, we found that *FKBP5*rs3800373 genetic risk was not statistically significantly associated with CMP (β = −0.003, p = 0.701; Supplementary Table 2). We next examined the relationship between hippocampal volume and CMP severity. Because previous literature has indicated that the left and right hippocampi play different roles in the development of pain^24,26,46^, and because these two brain regions had different mean volumes (t = 44.095, p = 2.2 × 10^–16^), we assessed for associations between these brain regions and CMP severity independently. As shown in Supplementary Table 3, we found that neither left (β = −0.005, p = 0.238) nor right (β = −0.007, p = 0.130) hippocampal volumes were directly associated with CMP.

### A statistically significant interaction between *FKBP5*rs3800373 genetic risk and right hippocampal brain volume was associated with CMP severity in UKBB study participants

**Figure 1:**
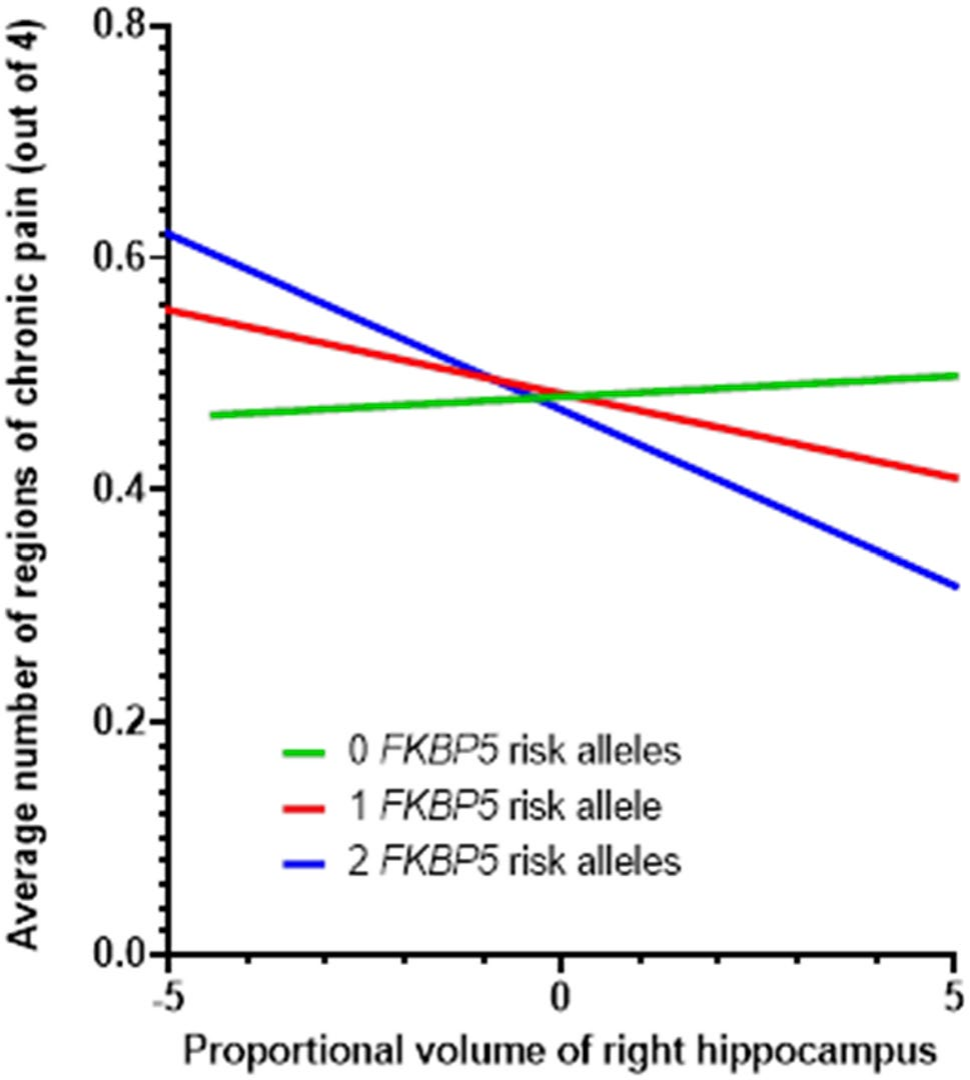
The number of *FKBP5*rs3800373 risk alleles moderates the relationship between the right hippocampus and CMP severity. Shown is the relationship between right hippocampal brain volume and chronic multisite musculoskeletal pain (CMP) severity in individuals from the UKBB study cohort (n = 36,822). Right hippocampal brain volume was adjusted for head size and standardized (mean = 0, SD = 1), see “Methods”. Individuals with two copies of the *FKBP5rs*3800373 risk allele (n = 3005) are represented by the blue line, individuals with one copy of the *FKBP5rs3800373* risk allele (n = 15,327) are represented by the red line, and individuals with no risk alleles are represented by the green line (n = 18,490). As shown in Supplementary Table 4, relationships represented by the blue and red lines were statistically significant (p < 0.05).

We next assessed our third hypothesis, that the interaction between *FKBP5*rs3800373 and hippocampal brain volume is associated with CMP severity. Here, as shown in Table 2, we found that the interaction between *FKBP5*rs3800373 genetic risk and right hippocampal brain volume (β = −0.020, p_raw_ = 0.002, p_adj_ = 0.01), but not left hippocampal brain volume (β = −0.015, p_raw_ = 0.023, p_adj_ = 0.12) was statistically significantly associated with CMP. The interaction explained 0.01% of the variance in CMP. Further, as shown in Fig. 1, we identified a dose-dependent inverse relationship between the number of *FKBP5*rs3800373 risk alleles and the relationship between right hippocampal brain volume on CMP severity such that individuals with two *FKBP5*rs3800373 risk alleles showed the strongest inverse relationship between right hippocampal volume and CMP (i.e. smaller right hippocampal volume was associated with increased CMP severity; β = −0.031, p = 0.045; Supplementary Table 4). In those individuals with one *FKBP5*rs3800373 risk allele, there was a less strong but statistically significant inverse relationship between right hippocampal volume and CMP (β = −0.016, p = 0.022). Finally in individuals with zero *FKBP5*rs3800373 risk alleles, we detected no statistically significant relationship between right hippocampal volume and CMP (β = 0.004, p = 0.639).

**Table 2:** Linear regression analyses assessing the interaction between *FKBP5* genetic risk, using the tagging allele rs3800373, and left and right hippocampal volumes on chronic multisite musculoskeletal pain in the UK Biobank (n = 36,822).

	Left hippocampus			Right hippocampus			
	β	S.E.	p value		β	S.E.	p value
Intercept	−0.302	0.047	< 0.001		−0.299	0.047	< 0.001
*FKBP5*rs3800373	−0.003	0.007	0.692		−0.003	0.007	0.694
Hippocampal volume	0.003	0.006	0.638		0.004	0.006	0.476
Age	0.001	0.0006	0.057		0.001	0.0006	0.065
Sex	−0.116	0.009	< 0.001		−0.117	0.009	< 0.001
BMI	0.029	0.001	< 0.001		0.029	0.001	< 0.001
Genotyping array	0.011	0.014	0.461		0.011	0.014	0.444
Imaging center (Reading)	−0.020	0.013	0.137		−0.020	0.013	0.133
Imaging center (Newcastle)	0.026	0.010	0.012		0.026	0.010	0.013
*FKBP5*rs3800373* Hippocampal volume	−0.015	0.007	0.023		−0.020	0.007	0.002

Chronic multisite musculoskeletal pain was defined as the number of musculoskeletal sites with pain that persisted for more than 3 months, ranging from 0 to 4. An additive genetic model was used to assess the effect of rs3800373. Genetic principal components 1–10 and ethnic background were included as covariates but omitted from the table for brevity.

### Secondary analyses identify childhood trauma as a moderator of the interaction between *FKBP5*rs3800373 genetic risk and right hippocampal brain volume on CMP

**Figure 2:**
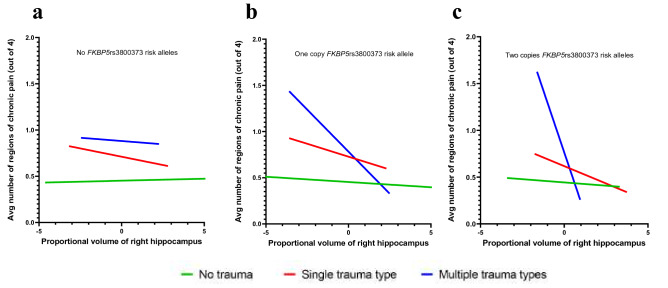
Childhood trauma and *FKBP5*rs3800373 risk alleles moderate the relationship between the right hippocampus and CMP severity. Shown is the relationship between right hippocampal brain volume and chronic multisite musculoskeletal pain (CMP) severity in individuals from the UK Biobank study with **(a)** 0, **(b)** 1, and **(c)** 2 *FKBP5*rs3800373 risk alleles and varying degrees of childhood trauma (n = 25,280). Right hippocampal brain volume was adjusted for head size and standardized (mean = 0, SD = 1), see “Methods”. Individuals who did not often experience childhood trauma are represented by the green line (“no trauma”), individuals who often experienced one type of childhood trauma are represented by the red line (“single trauma type”), and individuals who often experienced multiple (> 2) types of childhood trauma are represented by the blue line (“multiple trauma types”). As shown in Supplementary Table 8, the interaction between *FKBP5*rs3800373, right hippocampal volume, and childhood trauma was statistically significant (p < 0.05).

Numerous previous studies have identified significant gene-by-environment interactions between *FKBP5* risk alleles and significant stressors^47,48^. Therefore, in extension to the above findings, via secondary analyses, we assessed whether distress in the form of early childhood trauma moderates the relationship between *FKBP5*rs3800373/right hippocampal volume and CMP (Supplementary Fig. 2). We performed this analysis in a sub-cohort of UKBB participants with available childhood trauma data (n = 25,280). Because this subset only included a portion of the full cohort used for genetic and brain volume analyses, we first assessed whether the above identified relationships remained significant in the sub-cohort. Consistent with the above findings, in this sub-cohort we found no direct association between *FKBP5*rs3800373 genetic risk and CMP (β = −0.001, p = 0.853; Supplementary Table 5), between left hippocampal brain volume and CMP (β = −0.005, p = 0.401; Supplementary Table 6), or between right hippocampal brain volume and CMP (β = −0.006, p = 0.285; Supplementary Table 6). Additionally, consistent with above results, we identified a statistically significant interaction between *FKBP5*rs3800373 genetic risk and right hippocampal volume on CMP (β = −0.017, p = 0.032; Supplementary Table 7). We then examined the interaction between childhood trauma severity and right hippocampal volume on CMP in participants with 0, 1, or 2 *FKBP5*rs3800373 risk alleles. The interaction was statistically significant in participants with 1 (β = −0.084, p = 0.006; Table 3) or 2 (β = −0.168, p = 0.042; Table 3) *FKBP5*rs3800373 risk alleles such that in individuals with higher levels of childhood trauma and *FKBP5*rs3800373 genetic risk, decreased hippocampal volume was associated with increased CMP (Fig. 2). For individuals with 1 or 2 risk alleles, the interaction explained 0.58% and 0.30% of the variance in CMP, respectively. No interaction between the level of childhood trauma and right hippocampal volume was observed in those without an *FKBP5*rs3800373 risk allele (β = −0.020, p > 0.05; Table 3). Further, when we assessed an interactive relationship between these three factors, right hippocampal volume, *FKBP5*rs3800373 genetic risk, and childhood trauma severity on CMP, we identified a statistically significant relationship between the interaction of these three factors and CMP (β = −0.064, p = 0.047; Supplementary Table 8) that persisted when accounting for symptoms of anxiety and depression (β = −0.081, p = 0.016; Supplementary Table 9). Of note, because a second functional SNP, *FKBP5*rs1360780 (linkage disequilibrium with *FKBP5*rs3800373, *D*′ = 0.89, *r*^2^ = 0.78) has been shown to be associated with childhood trauma and is functional, we also tested the triple interaction using *FKBP5*rs1360780 (β = −0.063, p = 0.048; Supplementary Table 10).

**Table 3:** Linear regression analyses assessing the interaction between childhood trauma and right hippocampal volume on chronic multisite musculoskeletal pain in individuals with 0, 1, or 2 *FKBP5*rs3800373 risk alleles (n = 25,280).

	0 risk alleles (n = 12,777)			1 risk allele (n = 10,464)			2 risk alleles (n = 2,039)		
	β	S.E.	*p*	β	S.E.	*p*	β	S.E.	*p*
Intercept	−0.311	0.078	< 0.001	−0.260	0.084	0.002	−0.301	0.188	0.110
Right hippocampal volume	0.004	0.008	0.577	−0.012	0.008	0.158	−0.014	0.019	0.452
Childhood trauma severity	0.192	0.028	< 0.001	0.189	0.032	< 0.001	0.178	0.067	0.008
Age	0.0008	0.001	0.399	0.0007	0.001	0.520	0.002	0.002	0.331
Sex	−0.103	0.014	< 0.001	−0.105	0.016	< 0.001	−0.078	0.035	0.027
BMI	0.029	0.002	< 0.001	0.027	0.002	< 0.001	0.022	0.004	< 0.001
Genotyping array	0.020	0.024	0.408	0.001	0.026	0.972	0.0347	0.059	0.427
Imaging center (Reading)	−0.029	0.022	0.177	0.017	0.024	0.489	0.034	0.054	0.528
Imaging center (Newcastle)	0.004	0.018	0.803	0.035	0.019	0.074	0.152	0.043	< 0.001
Right hippocampal volume*childhood trauma severity	−0.020	0.028	0.470	−0.084	0.031	0.006	−0.168	0.083	0.042

Chronic multisite musculoskeletal pain was defined as the number of musculoskeletal sites with pain that persisted for 3 months or more, ranging from 0 to 4 sites. Genetic principal components 1–10 and ethnic background were included as covariates but omitted from the table for brevity.

## Discussion

Here we show for the first time and using the largest sample size to date that includes genetic, neuroimaging, and chronic pain data, that vulnerability to CMP is highest in individuals with a combination of *FKBP5*rs3800373 genetic risk alleles and smaller right hippocampal brain volumes. Further, we show that increased exposure to childhood trauma further extends this biological vulnerability to CMP. Importantly, these associations were not accounted for by differences in age, sex, and BMI, nor were they accounted for by symptoms of depression or anxiety. Together, these findings add to the growing body of literature indicating that multiple factors, including important biological × environmental interactions, compound risk for CMP. In addition, this work provides an important foundational framework for subsequent mechanistic work that aims to identify causal relationships between the identified factors.

While the main effect relationships that were hypothesized between *FKBP5* genetic risk and CMP and between hippocampal volume and CMP did not hold up in the current set of analyses (as they had in previous studies of chronic pain^16,49–51^) the interaction between these two factors did show association with CMP. Since both *FKBP5* and the hippocampus are central to stress system functioning^19–23,52,53^, and the combination of these stress system mediators substantially increases vulnerability to CMP (especially in individuals with a history of substantial childhood stressors), it suggests that the physiological stress-response system is critical to the development of CMP. Such findings are important because they provide further support to existing literature indicating that therapeutic strategies aimed at reducing CMP might be more effective if targeted to stress system mediators vs tissue injury generators^54,55^.

The HPA axis stress system and its role in the development of neuropsychiatric disorders has been studied extensively previously^48^. Data from this field, in addition to nascent literature from the pain field, can be used to help generate further hypotheses to later test how the identified stress-associated vulnerability factors might be contributing to the pathogenesis of CMP. For instance, because the protein encoded by *FKBP5*, FKBP51, regulates the affinity of the glucocorticoid receptor (GR) to the glucocorticoid cortisol^53^, and the *FKBP5* risk allele causes an increase in FKBP51 synthesis (thus decreasing glucocorticoid sensitivity^30^), one could hypothesize that in tissues with a high density of glucocorticoid receptors (e.g., hippocampus^23^), that increased glucocorticoid resistance (e.g., in individuals with *FKBP5* genetic risk and in individuals that have been exposed to substantial stressors) could cause maladaptive changes to tissue morphology (such as tissue atrophy). Such glucocorticoid-induced tissue atrophy has been observed previously via studies assessing cortisol inhibition of cellular formation and proliferation in the hippocampus^56^, likely via apoptosis in the subgranular zone of the dentate gyrus^57^. One might then hypothesize that lower glucocorticoid sensitivity and atrophied hippocampal tissue (due to glucocorticoids) lead to smaller anatomical hippocampal sizes that predispose an individual to CMP via the reduced negative feedback inhibition control of the hippocampus on the HPA axis ^58–61^. Chronic pain poses an allostatic load on the system^62^, and elevated basal glucocorticoid cortisol in chronic pain patients with smaller hippocampal volumes have been previously observed^63^. Therefore, it is possible that glucocorticoid resistance in the hippocampus due to *FKBP5* genetic risk, reduces this negative feedback inhibition control, thus sustaining a maladaptive endocrine stress response, and exacerbating CMP. While substantial data, including the data presented here, supports these mechanistic hypotheses, the direct causal relationships between the identified vulnerability factors and CMP has yet to be studied.

In addition to hippocampal volume and *FKBP5* genetic risk being important to the development of CMP, we also showed in this study that childhood stress via emotional, physical, and/or sexual abuse increases an individual’s vulnerability to CMP. In extension to the above mechanistic hypotheses, one might further expand upon the hypotheses to include the role that childhood trauma plays in the pathogenic mechanisms driving CMP. For instance, evidence from multiple species indicates that traumatic events in childhood alters normal development of brain structure, function and behaviour^64^, most notably in the hippocampus^65–70^. Additionally, the severity of childhood trauma and the developmental timing of this trauma influence stress responses in adulthood such that children who suffered from physical or sexual abuse in the first five years of life were more likely to have HPA axis dysregulation^71^. These HPA axis changes due to childhood abuse are thought to be mediated by epigenetic changes such as DNA de-methylation of *FKBP5*^72^, which occurs more commonly in individuals with at least one copy of the genetic risk allele in this gene^42,47^. Finally, as might be relevant to individuals in the UK Biobank cohort (i.e. with an average age of ~ 64 in the current sub-cohort), previous studies indicate that the effect that childhood trauma has on FKBP51, the hippocampus, and HPA axis persists throughout the lifespan^72–75^.

Several limitations should be considered when interpreting these study results. First, the cross-sectional study design implemented here limited our ability to assess for direct causation (e.g. between an inciting traumatic event and subsequent transition from acute to chronic pain). Second, the UK Biobank cohort is comprised of mainly European/White individuals, thus the generalizability of our results to other racial/ethnic groups is not known. Third, we did not control for potential confounding by medication use (e.g. opioids) or by comorbid disorders other than frequently co-occurring neuropsychiatric conditions. Fourth, the extent of both CMP and childhood trauma in this cohort was relatively low, limiting the sample size in some stratified analyses. Fifth, since our dataset did not include severity or duration of pain, our CMP variable has a more limited characterization of pain than other studies; however, we created our CMP variable using methodology that is consistent with other research that studies pain in the UK Biobank dataset^76,77^. Finally, the presented results remain to be validated in an independent dataset.

In conclusion, we presented novel evidence that both *FKBP5*rs3800373 genetic risk and childhood trauma moderate the relationship between hippocampal volume and CMP. While previous evidence highlighted these factors as independent predictors of chronic musculoskeletal pain, we found here that these factors interact such that individuals with a combination of these related risk factors have the highest risk for CMP. Future studies should investigate whether these factors not only indicate vulnerability for CMP but whether they also contribute to the underlying pathogenesis of disease. Altogether, these findings increase our understanding of risk factors for CMP and identify potential targets for therapeutic intervention within the stress axis pathway.

## Methods

### Study design and population

The UK Biobank is a population-based cohort of over 500,000 participants that was designed to improve the prevention, diagnosis, and treatment of chronic conditions. Between 2006 and 2010, all UK residents registered with the National Health Service, aged 40–69 years, and living within 25 miles of one of the 22 assessment centers across the country, were invited to participate. At baseline assessment, participants completed a detailed touch-screen questionnaire which collected demographic, lifestyle, and medical information. Biological samples (such as blood and saliva) and physical measurements (such as height and weight) were also collected. Between 2014 and 2020, enrolled participants were invited to participate in a follow-up assessment in which they repeated the initial questionnaire and underwent brain imaging protocols. Participants also regularly responded to web-based questionnaires including a mental health questionnaire between 2016 and 2017. The current study includes 36,822 participants with genetic, brain imaging, and chronic pain data. A sub-cohort of 25,280 participants with childhood trauma data was used for secondary analyses.

The present analyses were conducted under UK Biobank application number 48168^45^. All participants provided informed consent and all methods described in the current manuscript were performed in accordance with the relevant guidelines and regulations. Further information on the consent procedure can be found elsewhere (http://biobank.ctsu.ox.ac.uk/crystal/field.cgi?id=200).

### Assessments

#### Chronic multisite musculoskeletal pain (CMP)

At the follow-up visit between 2014 and 2020 (at the same time that brain imaging data was acquired), participants were asked “In the last month have you experienced any of the following that interfered with your usual activities?” The response options were seven specific sites of pain (headache, facial pain, neck or shoulder pain, back pain, stomach or abdominal pain, hip pain, and knee pain) as well as “pain all over the body”, “none of the above”, or “prefer not to say.” Participants were permitted to select multiple painful sites unless they reported having “pain all over the body,” “none of the above”, or “prefer not to say.” Participants who indicated that they had pain at specific sites were then asked whether the pain at each site had been present for more than 3 months^78^. Consistent with previous studies^76,77^, only the neck or shoulder, back, hip, and knee sites were used to investigate musculoskeletal pain. Participants who reported having pain all over the body were excluded since chronic widespread pain is distinct from chronic musculoskeletal pain^79,80^. Chronic multisite musculoskeletal pain (CMP) was defined as the number of musculoskeletal sites with pain that persisted for more than 3 months, ranging from 0 to 4.

#### Hippocampal brain volume

Brain images were acquired using Siemens Skyra 3 T scanners in UK Biobank’s imaging centers in Cheadle (n = 23,495), Reading (n = 4491), and Newcastle (n = 8836) using identical acquisition protocols across sites^81^. T1-weighted brain images were processed using FreeSurfer v6.0 to automatically estimate right and left hippocampal volumes and total intracranial volumes^82–84^. Hippocampal volumes were divided by total intracranial volume in order to adjust for head size^85,86^. Right and left hippocampal volumes were normally distributed both before and after adjustment (Supplementary Fig. 1). We used a t-test to compare the difference in means (mean volume of right hippocampus = 3795 mm^3^, mean volume of left hippocampus = 3677 mm^3^; adjusted mean volume of right hippocampus = 0.00246 and the adjusted mean volume of left hippocampus = 0.00238). Standardized adjusted hippocampal volumes (mean = 0, SD = 1) were used in statistical models.

#### *FKBP5* genetic risk

Genotyping was performed by the UK Biobank on the full cohort. In the current study, we utilized genotyping data from only those participants with brain imaging data. Genotyping was performed using the Affymetrix UK BiLEVE Axiom array (n = 3469) and the Affymetrix UK Biobank Axiom array (n = 33,353). Quality control procedures, as described previously^87^, were performed centrally by UK Biobank personnel prior to distribution to individual investigators. In the current set of analyses we focused on a single genetic variant in the *FKBP5* gene, rs3800373, based on its ability to tag the main risk haplotype^88^ and because it has been shown previously to be associated with persistent musculoskeletal pain via its allele-specific influence on microRNA regulation of *FKBP5* expression^18,49^. This allele was in Hardy–Weinberg equilibrium (p > 0.05) and had an excellent call rate (> 99%). Consistent with the reported minor allele frequency (MAF) of rs38003873 for the ‘British in England and Scotland’ (GBR) sub-population of European (EUR) ancestry (MAF = 32%, 1000 Genomes), the MAF for participants included in the current study was 29%. An additive genetic model (homozygous for the major allele (AA) = 0, heterozygous (CA) = 1, and homozygous for the minor allele (CC) = 2) was used to assess the relationship between *FKBP5*rs3800373, hippocampal brain volume, childhood trauma and CMP. Of note, rs1360780 is in high linkage disequilibrium with rs3800373 (*D*′ = 0.89, *r*^2^ = 0.78), is associated with childhood trauma and has been shown to influence *FKBP5* transcription via DNA methylation^42^. Due to high LD between this allele and *FKBP5*rs3800373, we used this allele to confirm key findings demonstrated with *FKBP5*rs3800373.

#### Childhood trauma

A sample of the original cohort (n = 25,280) completed a subsequent online mental health questionnaire between 2016 and 2017 that contained questions pertaining to childhood trauma. Three questions from the Childhood Trauma Questionnaire^89^ were used to identify participants who experienced physical abuse (“When I was growing up…People in my family hit me so hard that it left me with bruises or marks”), emotional abuse (“When I was growing up…I felt that someone in my family hated me”), and sexual abuse (‘When I was growing up…someone molested me (sexually)”). Possible responses were never true, rarely true, sometimes true, often true, very often true, or prefer not to say. For each question, similarly to previous definitions of frequency^90^, participants who responded “often true” or “very often true” were said to frequently experience physical, emotional, or sexual abuse. Of those individuals frequently experiencing these childhood traumas, the count of childhood trauma types, ranging from 0 to 3, was calculated and used to capture the frequency and dimensions of childhood trauma that participants experienced.

#### Depression and anxiety symptoms

To control for neuropsychiatric conditions related to CMP, two additional items from the online mental health questionnaire were used to assess core symptoms of depression and anxiety. The question “Have you ever had a time in your life when you felt sad, blue, or depressed for two weeks or more in a row?” was used to assess lifetime depressive symptoms and the question “Have you ever had a period lasting one month or longer when most of the time you felt worried, tense, or anxious?” was used to assess anxiety symptoms. If participants had an affirmative response to the item, we considered the participant affected by the neuropsychiatric condition.

### Statistical analyses

Sociodemographic characteristics were summarized using standard descriptive statistics. The relationship between *FKBP5*rs3800373, hippocampal brain volume, and chronic multisite musculoskeletal pain in the UK Biobank cohort was assessed using general linear models. Interactions between *FKBP5*rs3800373 and hippocampal volume were assessed by including corresponding product terms in the model. Based on previous analyses, age, sex, BMI, and self-reported ethnic background were included in models as covariates^77,91,92^. Models assessing the effect of *FKBP5*rs3800373 genetic risk also included genotyping array and top 10 genetic principal components as covariates. Models assessing the effect of hippocampal volume also included imaging site as a covariate, similarly to previous studies^86,92,93^. The coefficient of determination (R^2^, i.e. the proportion of the variation in the dependent variable that is predictable from the independent variable) of the interaction term was calculated as the difference between R^2^ of the full model that contains the interaction term along with covariates and the R^2^ of the null model that only includes covariates. We adjusted for multiple testing using Bonferroni correction and reported both raw and adjusted p-values. Statistical significance was determined based on adjusted p values. The sample (n = 36,822) was sufficient to test for gene-environment interactions. The minimum sample size to achieve 80% power at 5% significance level to detect a small effect gene-environment interaction for a genetically dominant SNP with p = 0.3 frequency is > 20,000 participants^94^.

Secondary analyses tested moderation via the interaction between childhood trauma and hippocampal volume on CMP in individuals with 0, 1, and 2 *FKBP5*rs3800373 risk alleles. All analyses were performed using RStudio server (1.4.1106-5) and all graphs were made for publication using GraphPad Prism v 9.1.2.

## Supplementary Information

Supplementary Information.
