# Supplementary material for: Hippocampal volume, FKBP5 genetic risk alleles, and childhood trauma interact to increase vulnerability to chronic multisite musculoskeletal pain
Source: Sci Rep. 2022 Apr 20;12:6511. doi: 10.1038/s41598-022-10411-9 (PMC9021300; doi:10.1038/s41598-022-10411-9)
Supplement: Supplementary file 1 — Supplementary Information. [file 41598_2022_10411_MOESM1_ESM.docx]

**Hippocampal volume, *FKBP5* genetic risk alleles, and childhood trauma interact to increase vulnerability to chronic multisite musculoskeletal pain**

**a**

**b**


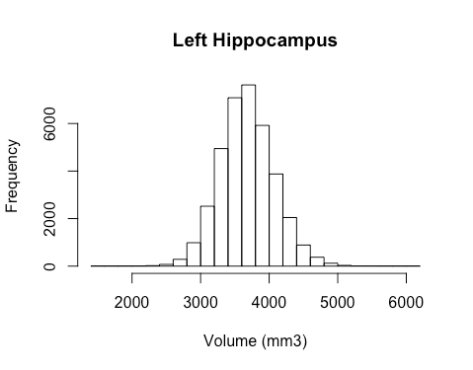

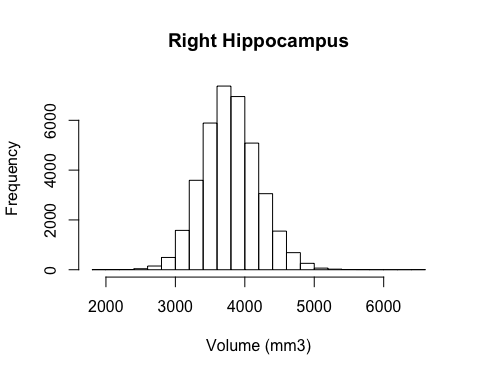


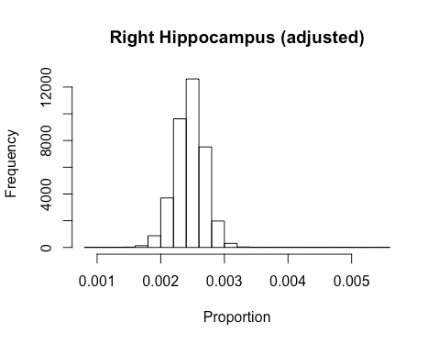

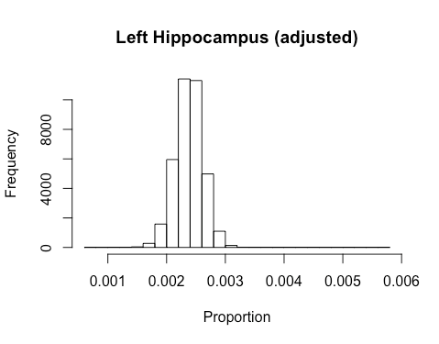


**c**

**d**

**Supplementary Figure 1**. Distribution of the left and right hippocampal volumes before (a and b) and after (c and d) adjustment for intracranial volume.

| **Supplementary Table 1.** Average number of sites and quantity of sites of chronic multisite musculoskeletal pain (CMP) in UK Biobank participants included in the current study (n=36,822). | |
| --- | --- |
| Average number of sites of CMP | 0.48 (0.81) |
| No CMP (0 sites), n (%) | 24,784 (67.3) |
| 1 site of CMP | 7,896 (21.4) |
| 2 sites of CMP | 2,919 (7.9) |
| 3 sites of CMP | 936 (2.6) |
| 4 sites of CMP | 287 (0.8) |
| Possible sites of CMP included neck or shoulder, back, hip, and knee sites. | |

| **Supplementary Table 2.** Linear regression analysis assessing the relationship between *FKBP5* genetic risk using tagging allele *FKBP5*rs3800373 and chronic multisite musculoskeletal pain in the subset of the UK Biobank cohort used in the current study (n=36,822). | | | |
| --- | --- | --- | --- |
|  | **β** | **S.E.** | **p value** |
| Intercept | -0.319 | 0.045 | <0.001 |
| *FKBP5*rs3800373 | -0.003 | 0.007 | 0.701 |
| Age | 0.001 | 0.0006 | 0.0132 |
| Sex | -0.114 | 0.008 | <0.001 |
| BMI | 0.029 | 0.001 | <0.001 |
| Genotyping array | 0.010 | 0.014 | 0.466 |
| Study site (Reading) | -0.019 | 0.013 | 0.149 |
| Study site (Newcastle) | 0.026 | 0.010 | 0.013 |
| Chronic multisite musculoskeletal pain was defined as the number of musculoskeletal sites with pain that persisted for more than 3 months, ranging from 0 to 4. An additive genetic model was used to assess the effect of rs3800373. Genetic principal components 1-10 and ethnic background were included as covariates but omitted from the table for brevity. | | | |

| **Supplementary Table 3.** Linear regression analyses assessing the relationship between left and right hippocampal volumes and chronic multisite musculoskeletal pain in all individuals with available neuroimaging data in the UK Biobank study (n=36,822). | | | | | | |
| --- | --- | --- | --- | --- | --- | --- |
|  | **Left Hippocampus** | | | **Right Hippocampus** | | |
|  | **β** | **S.E.** | **p value** | **β** | **S.E.** | **p value** |
| Intercept | -0.300 | 0.046 | <0.001 | -0.297 | 0.046 | <0.001 |
| Hippocampal volume | -0.005 | 0.0046 | 0.238 | -0.007 | 0.005 | 0.130 |
| Age | 0.001 | 0.0006 | 0.058 | 0.001 | 0.0006 | 0.066 |
| Sex | -0.116 | 0.009 | <0.001 | -0.117 | 0.009 | <0.001 |
| BMI | 0.029 | 0.001 | <0.001 | 0.029 | 0.001 | <0.001 |
| Study site (Reading) | -0.022 | 0.013 | 0.095 | -0.022 | 0.013 | 0.094 |
| Study site (Newcastle) | 0.028 | 0.010 | 0.005 | 0.028 | 0.010 | 0.005 |

| **Supplementary Table 4.** Stratified linear regression analyses assessing the relationship between right hippocampal volume and chronic multisite musculoskeletal pain in individuals with 0, 1, or 2 *FKBP5*rs3800373 risk alleles in the UK Biobank (n=36,822). | | | | | | | | | |
| --- | --- | --- | --- | --- | --- | --- | --- | --- | --- |
|  | **0 risk alleles (n=18,490)** | | | **1 risk allele (n=15,327)** | | | **2 risk alleles (n=3,005)** | | |
|  | **β** | **S.E.** | **p value** | **β** | **S.E.** | **p value** | **β** | **S.E.** | **p value** |
| Intercept | -0.342 | 0.066 | <0.001 | -0.239 | 0.072 | <0.001 | -0.351 | 0.161 | 0.029 |
| Right hippocampal volume | 0.004 | 0.006 | 0.540 | -0.016 | 0.007 | 0.022 | -0.031 | 0.016 | 0.045 |
| Age | 0.001 | 0.001 | 0.091 | 0.0004 | 0.001 | 0.641 | 0.002 | 0.002 | 0.265 |
| Sex | -0.123 | 0.012 | <0.001 | -0.114 | 0.013 | <0.001 | -0.087 | 0.030 | 0.004 |
| BMI | 0.030 | 0.001 | <0.001 | 0.028 | 0.001 | <0.001 | 0.026 | 0.003 | <0.001 |
| Genotyping array | 0.011 | 0.020 | 0.572 | 0.002 | 0.022 | 0.917 | 0.057 | 0.051 | 0.267 |
| Site (Reading) | -0.035 | 0.019 | 0.063 | -0.009 | 0.021 | 0.658 | 0.011 | 0.046 | 0.810 |
| Site (Newcastle) | 0.007 | 0.015 | 0.639 | 0.030 | 0.016 | 0.060 | 0.120 | 0.036 | <0.001 |
|  | | | | | | | | | |


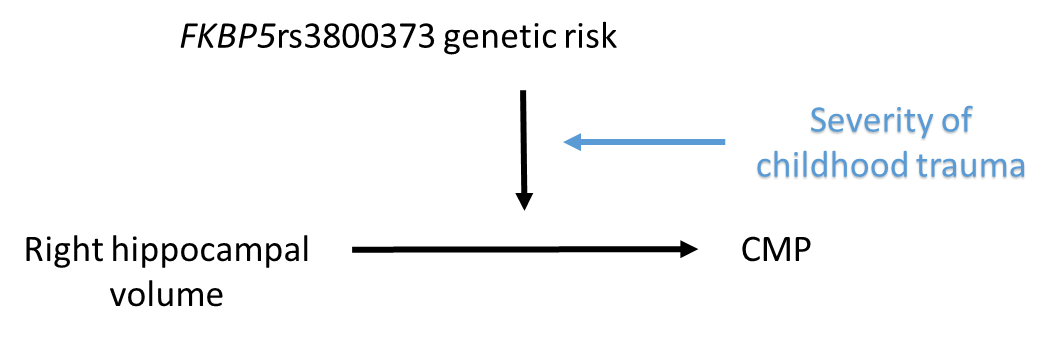


**Supplementary Figure 2**. Simplified diagram showing the relationship between factors assessed in the current study using UK Biobank cohort data. In primary analyses (black text and arrows), we showed that the relationship between right hippocampal volume and chronic multisite musculoskeletal pain (CMP) was moderated by *FKBP5*rs3800373. In secondary analyses (blue text and arrow), we assessed whether the severity of childhood trauma further moderates the relationships identified in primary analyses.

| **Supplementary Table 5.** Linear regression analysis assessing the relationship between *FKBP5*rs3800373 genetic risk and chronic multisite musculoskeletal pain in the cohort of individuals with available data on childhood trauma (n=25,280). | | | |  |
| --- | --- | --- | --- | --- |
|  | **β** | **S.E.** | **p value** | |
| Intercept | -0.294 | 0.053 | <0.001 | |
| rs3800373 | -0.001 | 0.008 | 0.853 | |
| Age | 0.001 | 0.0007 | 0.173 | |
| Sex | -0.105 | 0.010 | <0.001 | |
| BMI | 0.028 | 0.001 | <0.001 | |
| Genotyping array | 0.016 | 0.017 | 0.350 | |
| Site (Reading) | -0.004 | 0.002 | 0.812 | |
| Site (Newcastle) | 0.026 | 0.012 | 0.035 | |
| Chronic multisite musculoskeletal pain was defined as the number of musculoskeletal sites with pain that persisted for more than 3 months, ranging from 0 to 4. An additive genetic model was used to assess the effect of rs3800373. Genetic principal components 1-10 and ethnic background were included as covariates but omitted from the table for brevity. | | | |  |

| **Supplementary Table 6.** Linear regression analyses assessing the relationship between left and right hippocampal volumes and chronic multisite musculoskeletal pain in the cohort of individuals with available data on childhood trauma (n=25,280). | | | | | | | |
| --- | --- | --- | --- | --- | --- | --- | --- |
|  | **Left Hippocampus** | | | **Right Hippocampus** | | |  |
|  | **β** | **S.E.** | **p value** | **β** | **S.E.** | **p value** |  |
| Intercept | -0.279 | 0.055 | <0.001 | -0.277 | 0.055 | <0.001 |  |
| Hippocampal volume | -0.005 | 0.005 | 0.401 | -0.006 | 0.005 | 0.285 |  |
| Age | 0.001 | 0.001 | 0.312 | 0.001 | 0.001 | 0.331 |  |
| Sex | -0.107 | 0.010 | <0.001 | -0.107 | 0.010 | <0.001 |  |
| BMI | 0.028 | 0.001 | <0.001 | 0.028 | 0.001 | <0.001 |  |
| Site (Reading) | -0.008 | 0.015 | 0.592 | -0.008 | 0.015 | 0.590 |  |
| Site (Newcastle) | 0.028 | 0.012 | 0.020 | 0.028 | 0.012 | 0.020 |  |

| **Supplementary Table 7.** Linear regression analyses assessing the interaction between *FKBP5*rs3800373 genetic risk and left and right hippocampal volumes on chronic multisite musculoskeletal pain in the childhood trauma cohort (n=25,280). | | | | | | |
| --- | --- | --- | --- | --- | --- | --- |
|  | **Left Hippocampus** | | | **Right Hippocampus** | | |
|  | **β** | **S.E.** | **p value** | **β** | **S.E.** | **p value** |
| Intercept | -0.281 | 0.055 | <0.001 | -0.279 | 0.055 | <0.001 |
| *FKBP5*rs3800373 | -0.001 | 0.008 | 0.853 | -0.001 | 0.008 | 0.855 |
| Hippocampal volume | 0.003 | 0.007 | 0.718 | 0.004 | 0.007 | 0.611 |
| Age | 0.001 | 0.001 | 0.311 | 0.001 | 0.001 | 0.333 |
| Sex | -0.107 | 0.010 | <0.001 | -0.108 | 0.010 | <0.001 |
| BMI | 0.028 | 0.001 | <0.001 | 0.028 | 0.001 | <0.001 |
| Genotyping array | 0.016 | 0.017 | 0.350 | 0.017 | 0.017 | 0.341 |
| Site (Reading) | -0.004 | 0.016 | 0.795 | -0.004 | 0.016 | 0.791 |
| Site (Newcastle) | 0.027 | 0.013 | 0.034 | 0.027 | 0.013 | 0.033 |
| *FKBP5*rs3800373*  Hippocampal volume | -0.013 | 0.008 | 0.103 | -0.017 | 0.008 | 0.032 |
| Chronic multisite musculoskeletal pain was defined as the number of musculoskeletal sites with pain that persisted for more than 3 months, ranging from 0 to 4. An additive genetic model was used to assess the effect of *FKBP5*rs3800373. Genetic principal components 1-10 and ethnic background were included as covariates but omitted from the table for brevity. | | | | | | |

| **Supplementary Table 8.** Linear regression analysis assessing the interaction between *FKBP5*rs3800373 genetic risk, right hippocampal volume, and childhood trauma on chronic multisite musculoskeletal pain in the childhood trauma cohort (n=25,280). | | | |
| --- | --- | --- | --- |
|  | **β** | **S.E.** | **p value** |
| Intercept | -0.293 | 0.055 | <0.001 |
| *FKBP5*rs3800373 | -0.001 | 0.008 | 0.934 |
| Right hippocampal volume | 0.004 | 0.007 | 0.547 |
| Childhood trauma | 0.195 | 0.026 | <0.001 |
| Age | 0.001 | 0.001 | 0.161 |
| Sex | -0.102 | 0.010 | <0.001 |
| BMI | 0.027 | 0.001 | <0.001 |
| Genotyping array | 0.013 | 0.017 | 0.430 |
| Site (Reading) | -0.004 | 0.016 | 0.780 |
| Site (Newcastle) | 0.029 | 0.013 | 0.021 |
| *FKBP5*rs3800373*right hippocampal volume | -0.014 | 0.008 | 0.079 |
| *FKBP5*rs3800373*childhood trauma | -0.009 | 0.030 | 0.763 |
| right hippocampal volume*childhood trauma | -0.021 | 0.026 | 0.428 |
| *FKBP5*rs3800373*right hippocampal volume*childhood trauma | -0.064 | 0.032 | 0.047 |
| Chronic multisite musculoskeletal pain was defined as the number of musculoskeletal sites with pain that persisted for more than 3 months, ranging from 0 to 4. An additive genetic model was used to assess the effect of rs3800373. Genetic principal components 1-10 and ethnic background were included as covariates but omitted from the table for brevity. | | | |

| **Supplementary Table 9.** Linear regression analysis assessing the interaction between *FKBP5*rs3800373 genetic risk, right hippocampal volume, and childhood trauma on chronic multisite musculoskeletal pain in the childhood trauma cohort, controlling for chronic depression and chronic anxiety (n=23,852). | | | |
| --- | --- | --- | --- |
|  | **β** | **S.E.** | **p value** |
| Intercept | -0.414 | 0.056 | <0.001 |
| *FKBP5*rs3800373 | -0.002 | 0.008 | 0.773 |
| Right hippocampal volume | 0.003 | 0.007 | 0.671 |
| Childhood trauma | 0.155 | 0.027 | <0.001 |
| Age | 0.002 | 0.001 | 0.010 |
| Sex | -0.069 | 0.011 | <0.001 |
| BMI | 0.026 | 0.001 | <0.001 |
| Genotyping array | 0.003 | 0.017 | 0.872 |
| Site (Reading) | -0.009 | 0.016 | 0.581 |
| Site (Newcastle) | 0.026 | 0.013 | 0.038 |
| Chronic depression | 0.001 | 0.011 | <0.001 |
| Chronic anxiety | 0.103 | 0.012 | <0.001 |
| *FKBP5*rs3800373*right hippocampal volume | -0.013 | 0.008 | 0.092 |
| *FKBP5*rs3800373*childhood trauma | -0.020 | 0.032 | 0.520 |
| right hippocampal volume*childhood trauma | -0.011 | 0.027 | 0.695 |
| *FKBP5*rs3800373*right hippocampal volume*childhood trauma | -0.081 | 0.033 | 0.016 |
| Chronic multisite musculoskeletal pain was defined as the number of musculoskeletal sites with pain that persisted for more than 3 months, ranging from 0 to 4. An additive genetic model was used to assess the effect of rs3800373. Genetic principal components 1-10 and ethnic background were included as covariates but omitted from the table for brevity. | | | |

| **Supplementary Table 10.** Linear regression analysis assessing the interaction between *FKBP5*1360780 genetic risk, right hippocampal volume, and childhood trauma on chronic multisite musculoskeletal pain in the childhood trauma cohort (n=25,241). | | | |
| --- | --- | --- | --- |
|  | **β** | **S.E.** | **p value** |
| Intercept | -0.294 | 0.055 | <0.001 |
| *FKBP5*1360780 | -0.002 | 0.008 | 0.807 |
| Right hippocampal volume | 0.005 | 0.007 | 0.486 |
| Childhood trauma | 0.189 | 0.027 | <0.001 |
| Age | 0.001 | 0.001 | 0.160 |
| Sex | -0.102 | 0.010 | <0.001 |
| BMI | 0.028 | 0.001 | <0.001 |
| Genotyping array | 0.013 | 0.017 | 0.427 |
| Site (Reading) | -0.004 | 0.016 | 0.777 |
| Site (Newcastle) | 0.029 | 0.013 | 0.020 |
| *FKBP5*1360780*right hippocampal volume | -0.014 | 0.008 | 0.064 |
| *FKBP5*1360780*childhood trauma | -0.004 | 0.030 | 0.883 |
| right hippocampal volume*childhood trauma | -0.021 | 0.027 | 0.436 |
| *FKBP5*1360780*right hippocampal volume*childhood trauma | -0.063 | 0.032 | 0.048 |
| Chronic multisite musculoskeletal pain was defined as the number of musculoskeletal sites with pain that persisted for more than 3 months, ranging from 0 to 4. An additive genetic model was used to assess the effect of rs1360780. Genetic principal components 1-10 and ethnic background were included as covariates but omitted from the table for brevity. | | | |
